# Periwound Challenges Improve Patient Satisfaction in Wound Care

**DOI:** 10.1097/GOX.0000000000002134

**Published:** 2019-03-22

**Authors:** Apinut Wongkietkachorn, Palakorn Surakunprapha, Attapol Titapun, Nuttapone Wongkietkachorn, Supawich Wongkietkachorn

**Affiliations:** From the * Division of Plastic and Reconstructive Surgery, Department of Surgery, Faculty of Medicine, Mae Fah Luang University, Chiang Rai, Thailand; † Division of Plastic and Reconstructive Surgery, Department of Surgery, Faculty of Medicine, Khon Kaen University, Khon Kaen, Thailand; ‡ Department of Surgery, Faculty of Medicine, Khon Kaen University, Khon Kaen, Thailand; § Division of Plastic and Reconstructive Surgery, Department of Surgery, Q Clinic, Bangkok, Thailand; ¶ Department of Surgery, Faculty of Medicine, Thammasat University, Pathum Thani, Thailand

## Abstract

In wound care, we usually focus nearly all of our efforts on the wound area while paying little attention to the periwound area. Although the periwound area may seem unimportant, it matters to patients. A female patient was admitted with a wound at the perianal area. Wound dressing was performed using standard wet-to-dry gauzes. The patient had several small complaints including irritant contact dermatitis, skin maceration, pain during dressing change, and fecal contamination to the wound. In this case, we ended up switching to a different method of wound dressing. We went from using wet-to-dry gauzes for the primary dressing to a hydrofiber with silver dressing and from gauze and Micropore as a secondary dressing to an adhesive sodium carboxymethylcellulose foam dressing. This resolved all complaints. The patient’s satisfaction score using visual analog scale increased from 2 to 10 (out of 10 points). This example shows how even small details can make a significant difference in wound care. Because periwound care is often neglected, therapeutic algorithm that integrates major challenges in periwound care into wound healing strategies is proposed.

## Abstract 2

Supplemental Digital Content is available in the text.

In wound care, we usually focus nearly all of our efforts on the wound area while paying little attention to the periwound area. Although the periwound area may seem unimportant, it matters to patients. Thus, if we were to put more effort into periwound care, it could make a positive difference in terms of patient experience and satisfaction.^1^

Figures 1, 2 show a particularly difficult wound care case. The patient was admitted with a wound at the perianal area. Wound dressing was performed using standard wet-to-dry gauzes. In this case, the patient had several small complaints regarding the wound that had not been attended to. First, she had irritant contact dermatitis caused by Transpore and Micropore (3M, USA). It was suggested that this could be due to irritation from the adhesive substance used in the tape, which was made of acrylate adhesive (Fig. 1).^2^ Second, there was a mild skin maceration at the border of the wound caused by the wet-to-dry gauzes (see **figure, Supplemental Digital Content 1**, which displays problems from standard wound care, http://links.lww.com/PRSGO/A993). Third, she experienced significant pain during dressing change (pain score: 7/10). Finally, she had difficulty in defecation, as the feces would contaminate the external layer of gauze and later penetrate wound bed. Table 1 summarized problems found in this patient, which reflected major pitfalls in periwound care. These details may seem insignificant as they do not hinder the healing of the wound. However, if more care is taken in this respect, physicians may be able to provide a better experience for the patient and improve patient satisfaction.

**Table 1.**
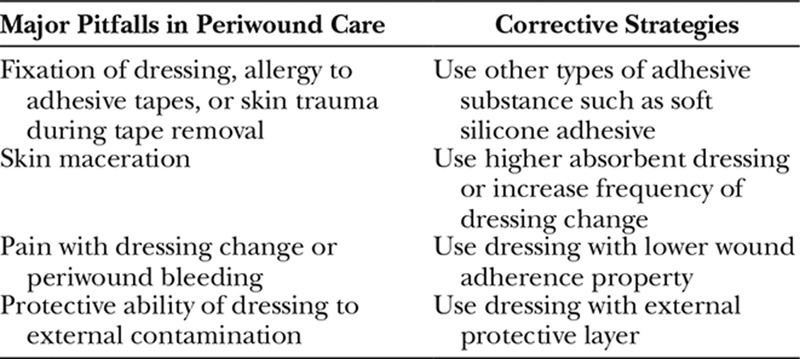
Major Pitfalls in Periwound Care and Corrective Strategies

**Fig. 1.**
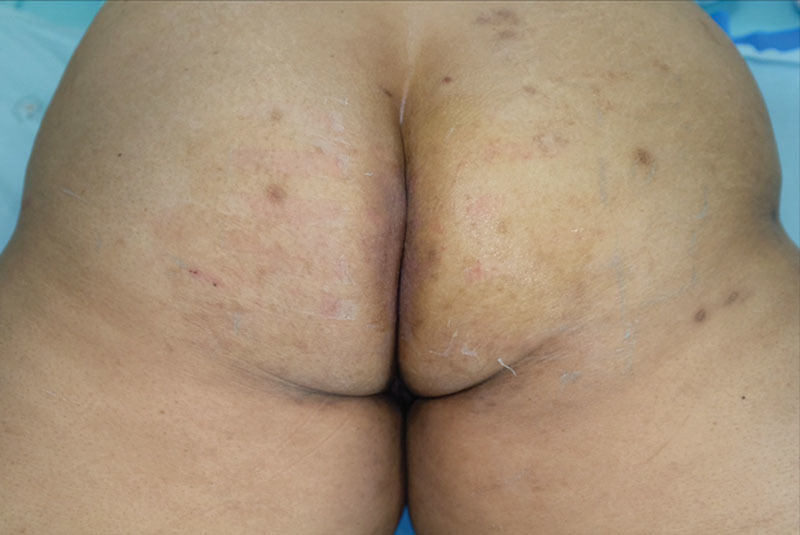
Standard wound care. The secondary dressing was gauze and Micropore, which caused irritant contact dermatitis and allowed feces to contaminate the wound bed.

**Fig. 2.**
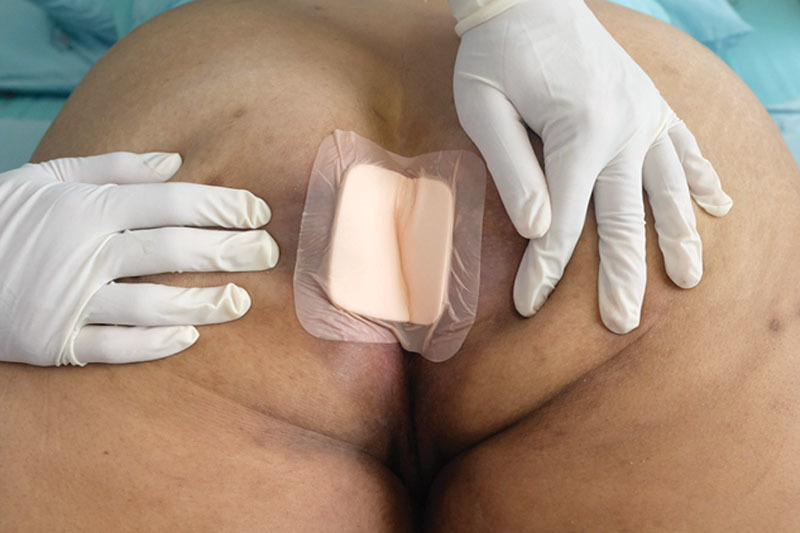
Wound care with careful attention to the minute details. The secondary dressing was a Na-CMC foam dressing (Adhesive Aquacel Foam). The irritant contact dermatitis was resolved.

In this case, we ended up switching to a different method of wound dressing. We went from using wet-to-dry gauzes for the primary dressing to a hydrofiber with silver dressing (Aquacel Ag^+^ Extra; ConvaTec, UK) and from gauze and Micropore as a secondary dressing to an adhesive sodium carboxymethylcellulose (Na-CMC) foam dressing (Adhesive Aquacel Foam; ConvaTec, USA). This resolved the irritant contact dermatitis, as the adhesive substance used in the adhesive Na-CMC foam was made from silicone (Fig. 2). The macerated skin was also resolved after 5 days due to the adequate absorbency of the Na-CMC foam. The foam combined with the exudate inside the wound area, becoming a soft gel that blocked the exudate from leaving the wound area.^3^ In addition, the patient’s pain score using visual analog scale was significantly reduced^4^ from 7 to 2. Finally, defecation was easier as the external top layer of the Na-CMC foam, which was made of polyurethane film, was waterproof and was able to prevent feces from penetrating the wound bed (see **figure, Supplemental Digital Content 2**, which displays results of wound care with careful attention to the minute details, http://links.lww.com/PRSGO/A994). The patient’s satisfaction score using visual analog scale increased from 2 to 10 (out of 10 points). Because periwound care is often neglected, therapeutic algorithm that integrates major challenges in periwound care into wound healing strategies^5–7^ is proposed in Figure 3.

**Fig. 3.**
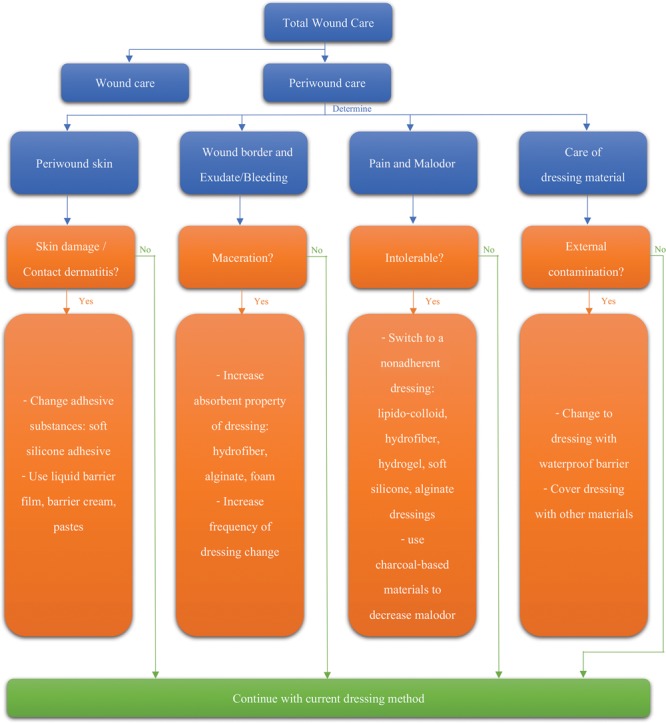
Therapeutic algorithm for major challenges in periwound care.

This example shows how even small details can make a significant difference in wound care.

## Supplementary Material
