# Supplementary material for: Periwound Challenges Improve Patient Satisfaction in Wound Care
Source: Plast Reconstr Surg Glob Open. 2019 Mar 22;7(3):e2134. doi: 10.1097/GOX.0000000000002134 (PMC6467635; doi:10.1097/GOX.0000000000002134)

Supplemental Digital Content 1 Problems from standard wound care. (a) The secondary dressing was gauze and Micropore™ (3M™, USA) which caused irritant contact dermatitis and allowed feces to contaminate the wound bed. (b) The primary dressing was wet-to-dry gauze, which resulted in mild skin maceration at the border of the wound and significant pain during dressing change.

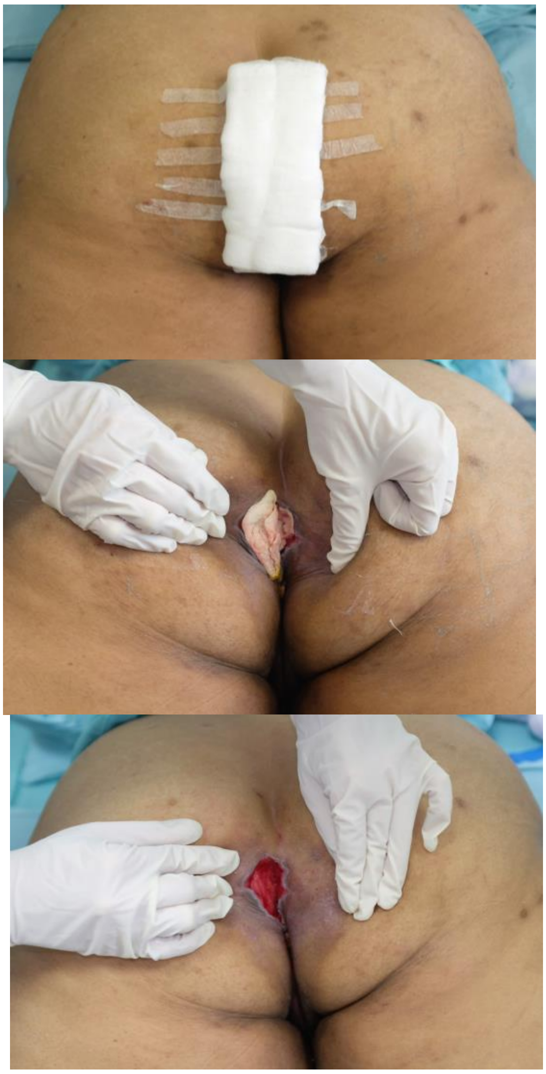

Supplement: Supplementary file 1 [file gox-7-e2134-s001.pdf]
