# Supplementary material for: Periwound Challenges Improve Patient Satisfaction in Wound Care
Source: Plast Reconstr Surg Glob Open. 2019 Mar 22;7(3):e2134. doi: 10.1097/GOX.0000000000002134 (PMC6467635; doi:10.1097/GOX.0000000000002134)

Supplemental Digital Content 2 Results of wound care with careful attention to the minute details. (a) The secondary dressing was a sodium carboxymethylcellulose (Na-CMC) foam dressing (Adhesive Aquacel® Foam; Convatec, USA). The irritant contact dermatitis was resolved. The foam external layer, which was made of polyurethane film, was waterproof and was able to prevent feces from penetrating the wound bed (b) The primary dressing was a hydrofiber with silver dressing (Aquacel® Ag^+ Extra™; Convatec, UK), which significantly decreased pain during dressing change. The Na-CMC foam also locked exudate inside the wound area and decreased skin maceration.

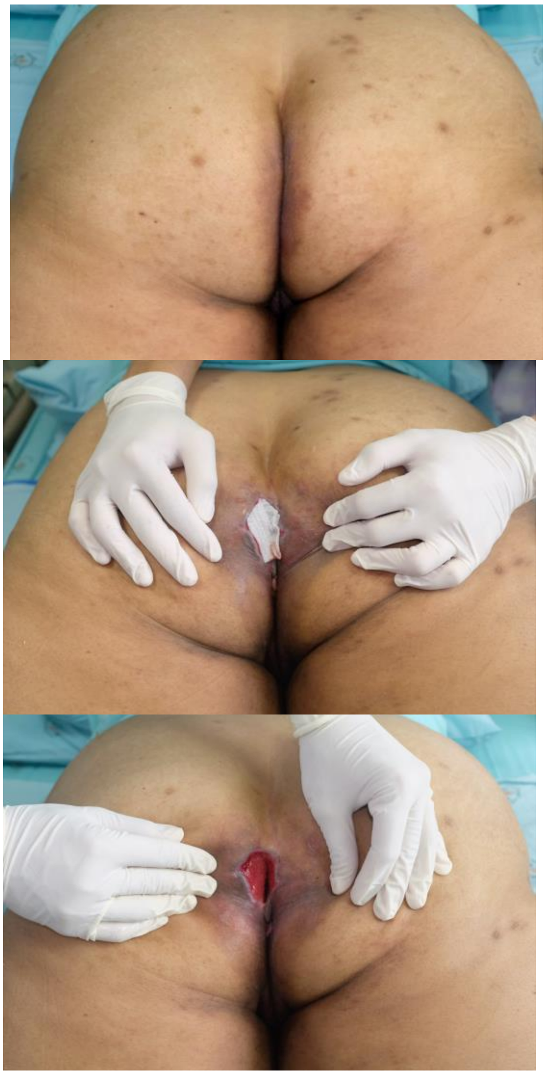

Supplement: Supplementary file 2 [file gox-7-e2134-s002.pdf]
